# Effect of Antibacterial Agents on Dentin Bond Strength of Bioactive Restorative Materials

**DOI:** 10.3390/polym15122612

**Published:** 2023-06-08

**Authors:** Roaa Abuljadayel, Nouf Aljadani, Hazim Almutairi, Alaa Turkistani

**Affiliations:** 1 Department of Restorative Dentistry, Faculty of Dentistry, King Abdulaziz University, P.O. Box 80209, Jeddah 21589, Saudi Arabia; rabuljadaiel@kau.edu.sa; 2 Faculty of Dentistry, King Abdulaziz University, P.O. Box 80209, Jeddah 21589, Saudi Arabia; naljedani0022@stu.kau.edu.sa (N.A.); halmutairi0091@stu.kau.edu.sa (H.A.)

**Keywords:** dentin, bioactive, composite resin, resin-modified glass ionomer, bond strength, chlorhexidine, silver diamine fluoride

## Abstract

Treating dentin surfaces with antibacterial agents prior to bonding bioactive restorations might affect their mechanical properties. In this study, we evaluated the effect of silver diamine fluoride (SDF) and chlorhexidine (CHX) on the shear bond strength (SBS) of bioactive restorative materials. Dentin discs were treated with SDF for 60 s or CHX for 20 s and bonded with four restorative materials, namely Activa Bioactive Restorative (AB), Beautifil II (BF), Fuji II LC (FJ), and Surefil One (SO). Control discs were bonded without treatment (n = 10). SBS was determined using a universal testing machine, and a scanning electron microscope (SEM) was used for the evaluation of the failure mode and the cross-sectional examination of adhesive interfaces. The SBS of each material between different treatments and of the different materials within each treatment were compared via a Kruskal–Wallis test. The SBS of AB and BF was significantly higher than that of FJ and SO in the control and CHX groups (*p* < 0.01). In the latter, SBS was higher in FJ than SO (*p* < 0.01). SO had a higher value with SDF compared to CHX (*p* = 0.01). The SBS of SDF-treated FJ was higher than that of the control (*p* < 0.01). SEM showed a more homogenous and improved interface of FJ and SO with SDF. Neither CHX nor SDF compromised the dentin bonding of bioactive restorative materials.

## 1. Introduction

Dental caries remains the most common oral disease worldwide. Caries lesions develop when a shift in the biological dynamic process in dental biofilm induces a chemical dissolution of dental tissues via bacterial acids. The efficient management of dental caries relies on early detection, accurate diagnosis, the prevention of disease progression, and minimal restorative intervention [1].

Minimal invasive dentistry (MID) is a modern approach to dental treatment that emphasizes the preservation of healthy tooth structure while effectively treating dental caries. The goal of MID is to achieve long-lasting restorations that require minimal intervention and have a low risk of secondary caries development [1,2]. Conservative cavity preparation is a key component of MID, which involves removing only the infected dentin while preserving the affected and healthy dentin to help maintain the structural integrity of the tooth and reduce the risk of pulp exposure. Conservative cavity preparation can be achieved using various techniques, such as air abrasion, caries removal with detecting dye and hand instruments, or laser-assisted caries removal [3]. The use of adhesive restorative materials, such as glass ionomer cements, is also an essential component of MID. These materials can help to create a strong bond with the tooth structure and reduce the risk of microleakage and secondary caries [2,3]. Dental plaque accumulates much more in resin-based restoration materials than in glass ionomer cements and amalgam. As a result, residual bacteria or invading bacteria along the tooth/restoration interface may cause recurrent and secondary caries and pulp irritation following therapy [1]. An antimicrobial action of the restorative system is desirable to reduce cariogenic bacterial colonization on the tooth/restoration interface and the proliferation of residual bacteria in the cavity following preparation. This effect can be achieved by including antimicrobial agents or antibacterial monomers in the dental material formulation [4,5], by modifying the material composition to include organic nanoparticles that decrease demineralization and enhance remineralization, or by using inorganic nanoparticles that actively suppress the action of oral microorganisms [6]. 

In recent years, researchers have investigated the use of natural chemicals such as epigallocatechin-3-gallate (EGCG) in dental materials. EGCG has been demonstrated to exhibit antibacterial, anti-inflammatory, and antioxidant activities [7]. Du, X. et al. discovered that an EGCG-enhanced dental adhesive exhibited high levels of antibacterial action and improved the bonding strength to dentin compared to a control adhesive [7]. Another study revealed that EGCG effectively reduced the growth of bacteria linked with a variety of oral infections, including dental caries- and periodontal disease-causing bacteria, such as *S. Mutans* (*S. Mutans*) and *Porphyromonas gingivalis*. EGCG is hypothesized to exert its therapeutic effects by breaking bacterial cell membranes and decreasing the action of enzymes involved in bacterial metabolism [8]. 

In addition, a variety of dental composites with nanoparticles, such as calcium fluoride, hydroxyapatite, bioactive glass, silver, zinc, magnesium, and copper oxides, have been introduced. Composite materials containing nanoparticles of calcium phosphate or calcium fluoride were shown to be highly effective against *S. Mutans* [9]. Silver-containing nanocomposites also exhibited significant antibacterial effects through the inhibition of *S. Mutans* activity [10]. A previous study reported that an aqueous solution of silver nanoparticles prepared via the reduction of silver nitrate exhibited long-term activity against several oral microorganisms [11].

Recently, the use of silver diamine fluoride (AgFH_6_N_2_, SDF) to prevent and arrest caries activity in both primary and permanent teeth has become increasingly popular [12,13]. SDF is an alkaline anticariogenic solution that combines silver’s antibacterial properties with the remineralizing ability of fluoride [14]. It is composed mainly of ammonia hydroxide, silver nitrate, and hydrofluoric acid. Since its development in 1960, SDF has been shown to be a cost-effective cariostatic agent. In 2014, the US Food and Drug Administration (FDA) approved the use of SDF as a desensitizing agent. It is also considered an alternative to traditional drilling and filling procedures for elderly and patients with special needs [12]. Furthermore, SDF has been used as a pretreatment prior to the placement of restorative material, preventing the formation of secondary caries [15]. 

In situations in which carious lesions progress, leading to irreversible weakening of the structure and surface collapse, lost dental tissue should be replaced with a restorative material to promote pulpal health and replace the form, function, and esthetics of the tooth. Minimal intervention necessitates the selective excavation of infected dentin and the preservation of remineralizable, low-bacterial-load-affected dentin [3]. Cleaning the cavity with an antimicrobial agent is recommended to control any viable bacteria remaining in dentinal tubules after selective caries excavation [16]. Chlorhexidine (C_22_H_30_CL_2_N_10_, CHX) is considered the most widely used cavity disinfectant [17,18]. CHX is a broad-spectrum antibacterial that inhibits Gram-negative and Gram-positive bacteria and therefore suppresses the growth of residual bacteria [19]. Previous studies have reported that CHX increases the bond strength of resin composites, preserves the collagen matrix, and prevents degradation at the resin–dentin interface [16,20]. However, other studies reported a decrease in immediate dentin bond strength and marginal sealing, which may influence the longevity of the restoration [18].

Restoration with bioactive materials has become popular in recent years [21,22]. Depending on their composition and intended application, dental materials can have a wide range of bioactivities. The ability of a material to interact with biological systems, such as cells and tissues, in a way that promotes favorable benefits or limits negative effects is referred to as bioactivity. Some common bioactivities of dental materials include antimicrobial, remineralization, regeneration, and direct chemical bonding properties [21]. However, a bioactive restorative material can be defined as any material that is able to restore lost minerals and repair enamel and dentin tissue damage [21,22]. When immersed in a simulated body fluid or a solution containing inorganic phosphate, the material can create hydroxyapatite crystals on the cavity surface [21]. The placement of a restorative material that combines esthetic and mechanical properties with antibacterial and remineralization functions complies to the concept of minimal invasiveness [3]. Several resin composites with resin matrix components or bioactive fillers that promote remineralization, inhibit bacterial growth, and control pH have been introduced [4,23]. These materials have been shown to be effective in decreasing demineralization and increasing the acid resistance of the tooth structure [23]. Despite the beneficial effects, the ion leaching of bioactive materials may compromise physicochemical properties and could induce insufficient mechanical properties.

Glass ionomer cement (GIC) is the most widely used bioactive material for direct restoration. It is known for the fluoride release and the recharge of its fluoroaluminosilicate fillers, together with its anticariogenic and remineralization potential at the tooth–material interface [13]. The addition of resinous content in resin-modified glass ionomer (RMGIC) has improved the mechanical and adhesion characteristics of GIC; however, the bonding strength values are still inferior to those of resin composites [24]. 

New restorative materials with claimed bioactive behavior and promising mechanical properties have recently been launched. Activa Bioactive Restorative was introduced as a bulk-fill material with bioactive fillers and a resin matrix and has natural tooth-related physical and chemical properties [25]. This dual-cure material comprises reactive ionomer glass fillers, a bioactive ionic resin matrix, and a shock-absorbing rubberized resin component [26]. It reacts to oral pH changes and releases significant amounts of calcium, phosphate, and fluoride, stimulating hydroxyapatite formation and remineralization. According to the manufacturer, fluoride ion release in Activa Bioactive Restorative is higher than that in GIC. It is also claimed that the added resin monomers improve its flexural strength, fracture toughness, and wear resistance compared to compomers and RMGIC [26,27]. 

Giomers are a new category of bioactive glass ionomer/composite hybrid resin. These materials are based on fillers created from the complete or partial reaction of GIC with polyalkenoic acids called pre-reacted glass ionomer (PRG) fillers incorporated into bis-GMA (bisphenol A glycidyl methacrylate) and TEGDMA (triethylene glycol dimethacrylate) resins [28]. These fillers can release and recharge fluoride, aluminum, sodium, strontium, borate, and silicate ions, which have been shown to neutralize bacteriogenic acids and inhibit secondary caries. Beautifil II is a light-cured, direct restorative material product of this category. Aside from the antimicrobial activity, the unique filler structure simulates the light diffusion and transmission properties of a natural tooth and therefore offers predictable esthetic outcomes. 

Surefil One is a dual-cure, self-adhesive composite hybrid that combines the self-adhesion property of GIC with resin composite properties. Cavities of any depth can be filled using Surefil One without the need for etching, bonding, or layering. The composition is based on a polyacrylic acid of high molecular weight functionalized with polymerizable groups (called MOPOS by the manufacturer), which is claimed to create a strong composite-like structure and a durable adhesion to the dental substrate. Amide-based crosslinkers that polymerize with all formulation components are included to generate a three-dimensional network with improved mechanical strength. The mixture also contains fillers, a certain amount of water, and agents for photopolymerization and chemopolymerization [26].

There are few reports in the literature about the mechanical properties of these newly launched materials [24,25,26,29,30]. In addition, previous studies reported the effect of treatment with CHX and SDF on the bonding of resin composite to dentin [16,20,31,32,33]. However, the effect on the newly introduced bioactive materials is still unclear.

Therefore, the aim of this study was to (i) compare the shear bond strength (SBS) of each of the four bioactive restorative materials, and to determine whether surface treatment with SDF or CHX would influence the bond strength and (ii) to compare the SBS of the different bioactive restorative materials among each surface treatment (SDF, CHX, and no treatment). The following null hypotheses were tested: (1) surface treatment with SDF or CHX does not affect the SBS of each individual restorative material, and (2) there is no difference in the SBS of the different restorative materials among each surface treatment groups.

## 2. Materials and Methods

### 2.1. Specimen Preparation

A total of 132 sound human premolar teeth was obtained from patients, as approved by the Institutional Ethics Committee of King Abdulaziz University (026-02-22, 10 March 2022), and the study was performed in accordance with the Declaration of Helsinki’s guidelines and regulations. Flat mid-coronal dentin discs of approximately 5 mm thickness were obtained by trimming roots and cusps at a right angle to the long axis of the tooth with a grinding and polishing machine (MetaServ 250 with VectorTM Power Head, Buehler Ltd., Evanston, IL, USA). Discs were then embedded in self-cure acrylic resin (Vertex Dental, Soesterberg, Netherlands) using a cylindrical plastic mold. The exposed dentin surface was further wet-polished to 1200 grit using silicon carbide (SiC) sheets. 

Specimens were randomly assigned to three main groups according to the type of surface treatment: SDF (Advantage Arrest, Elevate Oral Care, West Palm Beach, FL, USA), CHX (Gluco-chex 2%, Cerkamed, Stalowa Wola, Poland), or no treatment as the control. For the SDF group, the dentin surface was dried with a three-way syringe, and SDF was applied and allowed to soak in for 60 s before rinsing and drying [13]. For the CHX group, CHX was applied for 20 s and air-dried for 5 s [34]. After surface treatment, each group was further subdivided into four groups according to the type of restorative material (n = 10), i.e., Activa Bioactive Restorative (AB, Pulpdent Corp., Watertown, MA, USA), Beautifil II (BF, Shofu Corp., Tokyo, Japan), Fuji II LC (FJ, GC Corp., Tokyo, Japan), or Surefil One (SO, Dentsply Sirona, Konstanz, Germany). 

**Figure 1:**
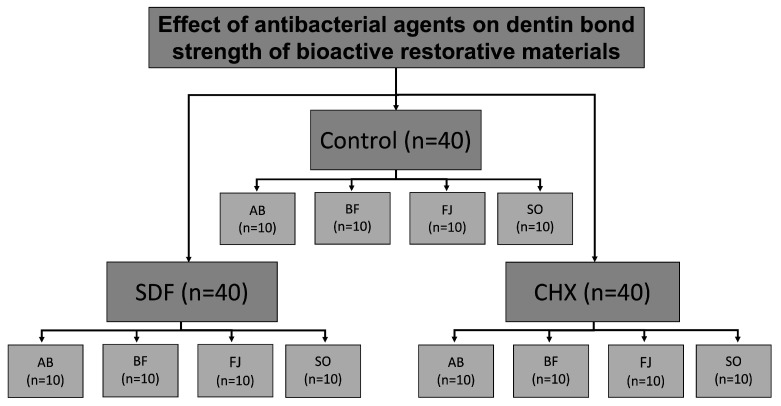
Flow chart of study design. AB; Activa Bioactive Restorative. BF: Beautifil II. FJ: Fuji II LC. SO: Surefil One. SDF: Silver Diamine Fluoride. CHX: Chlorhexidine.

In the ABF and BF groups, a universal bonding agent (Single Bond Universal, 3M ESPE, St. Paul, MN, USA) was applied in self-etch mode and cured using an LED curing unit (EliparTM Light Cure, 3M ESPE, St. Paul, MN, USA) at an intensity of 1200 mw/cm^2^ prior to the application of the restorative material [35]. Figure 1 summarizes specimen grouping, and Table 1 shows the composition and application of each material.

**Figure 2:**
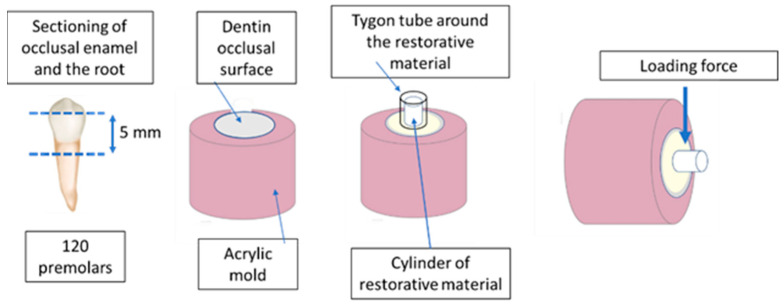
Schematic diagram of specimen preparation before a shear test was applied.

A Tygon tube (ququyi manufacturer, 3 mm × 3 mm “diameter × height”) was placed over the dentin surface and filled with the restorative materials according to the manufacturer’s instructions. Then, the tube was carefully removed using a sharp blade before testing was performed. Specimens were kept in 100% relative humidity for 24 h at 37 °C before testing was performed. A schematic diagram of specimen preparation is presented in Figure 2.

### 2.2. Shear Bond Testing

Blind specimens were mounted on a universal testing machine (Instron Corporation, Canton, MA, USA), and shear force was applied at the tooth–material interface to determine the fracture type and to calculate the SBS at the breaking force at a crosshead speed of 1 mm per second via a knife-edge blade using a 2 kN load cell. Shear strength was measured by calculating the load ratio of the shear failure (F in Newton) over the area of the material disc at the interface in mm^2^. The shear force at fracture was taken as the force that caused the specimen to debond [36].

### 2.3. Mode of Failure Observation

Following the test, debonded specimens were gold-sputter-coated and inspected under SEM (AURA100, Seron Technologies Inc., Uiwang-si, Republic of Korea) at a ×100 magnification to determine the mode of failure. The specimen’s surface was examined and appraised, and the type of failure was categorized as either an adhesive, cohesive, or mixed failure. The adhesive type of failure was identified as a failure that occurred in the adhesive or composite and was not related to dentin. Cohesive failure was considered a failure that took place entirely in dentin and resulted in a surface concavity. Failure that affected both the bonding or composite and dentin and gave the surface a mottled look was categorized as a mixed failure.

### 2.4. Statistical Analysis

The descriptive statistics of the SBS were presented as the mean with standard deviation and the median with interquartile ranges. The normality of SBS was assessed through visual inspection of a histogram and a Q–Q plot and by interpreting the results of skewness–kurtosis normality tests. The tests indicated the deviation of normality of the variable. Therefore, non-parametric tests were utilized.

The SBS of each bonding material between the different surface treatment groups were compared via a Kruskal–Wallis test. Results that were statistically significant were analyzed via multiple comparison tests (Wilcoxon rank-sum tests). Another similar analysis was carried out to compare the SBS of each surface treatment in regard to the different bonding materials. A *p*-value < 0.05 was considered statistically significant. The statistical analyses were performed using Stata version 12.1 software (StataCorp LP, College Station, TX, USA).

### 2.5. Scanning Electron Microscope (SEM)

Another three specimens were prepared from each study group. A 2 mm thick material buildup was bonded to dentin following the same protocol used earlier for the SBS test. Specimens were stored in distilled water at 37 °C for 24 h, then cross-sectioned through the bonded interface using a diamond saw. Specimens were then embedded in epoxy resin (Vertex Dental, Soesterberg, The Netherlands) and polished sequentially using 600–2000 SiC polishing sheets. The specimens were gold-sputter-coated, and a qualitative inspection under SEM (AURA100, Seron Technologies Inc., Uiwang-si, Republic of Korea) at ×1000 magnification was performed to visualize the bonding interface. 

## 3. Results

### 3.1. Shear Bond Testing

Table 2 presents a comparison of the SBS of each bonding material between the different surface treatments. For FJ, the SBS was higher with SDF surface treatment (median: 8.9; IQR: 6.6, 12.2) than with no surface treatment (median: 2.7; IQR: 1.5, 5.3). For SO, SBS was higher with SDF surface treatment (median: 8.4; IQR: 3.6, 10.7) than with CHX (median: 2.7; IQR: 2.2, 4.2). There was no difference in the SBS of AB and BF among the different surface treatments.

Table 3 illustrates the comparison of the SBS of the different bonding materials within each surface treatment groups. Among the CHX-treated specimens, FJ and SO had lower SBS than AB; furthermore, FJ and SO had lower SBS than BF, and SO had a lower SBS than FJ. Similarly, in the control group, FJ and SO had lower bond strength values than AB and BF.

### 3.2. Mode of Failure

**Figure 3:**
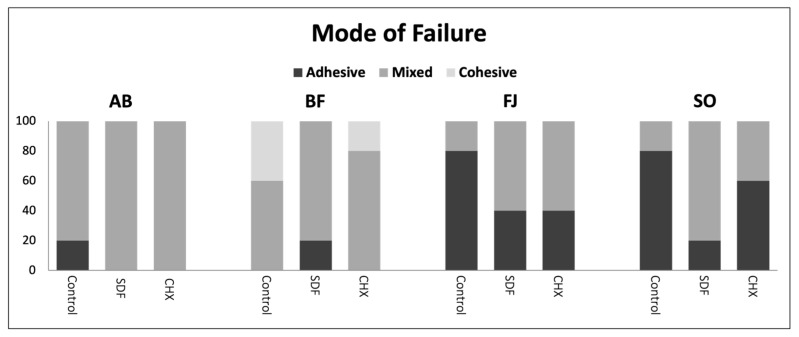
Bar graph showing the modes of failure in different test groups.

**Figure 4:**
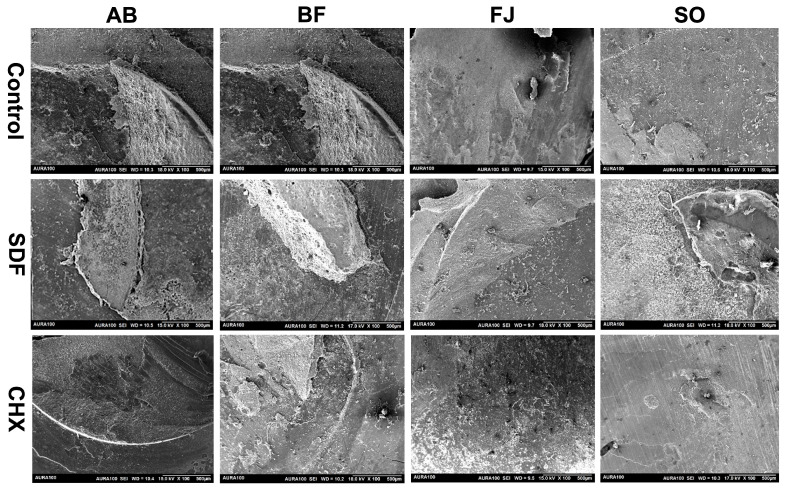
Representative SEM images of the modes of failure in different test groups.

Debonded specimens of different groups demonstrated different modes of failure. In control groups, the mode of failure in AB was 20% adhesive and 80% mixed. BF showed 60% mixed failure and 40% cohesive failure. FJ and SO, however, had exhibited 80% adhesive failure and 20% mixed failure, as shown in Figure 3 and Figure 4. SDF- and CHX-pretreated groups demonstrated a predominantly mixed type of failure, except for CHX-treated SO, which displayed mainly adhesive failure. In AB–SDF and AB–CHX, the mode of failure was 100% mixed. For SDF- and CHX-treated BF, the percentage of mixed failure was 80%, with 20% adhesive failure in SDF and cohesive failure in CHX. For FJ, both SDF- and CHX- treated groups displayed 40% adhesive failure and 60% mixed failure. When treated with SDF, the adhesive failure in SO decreased to 20%. However, with CHX treatment, the failure type was 60% adhesive and 40% mixed. 

### 3.3. SEM of the Interface

**Figure 5:**
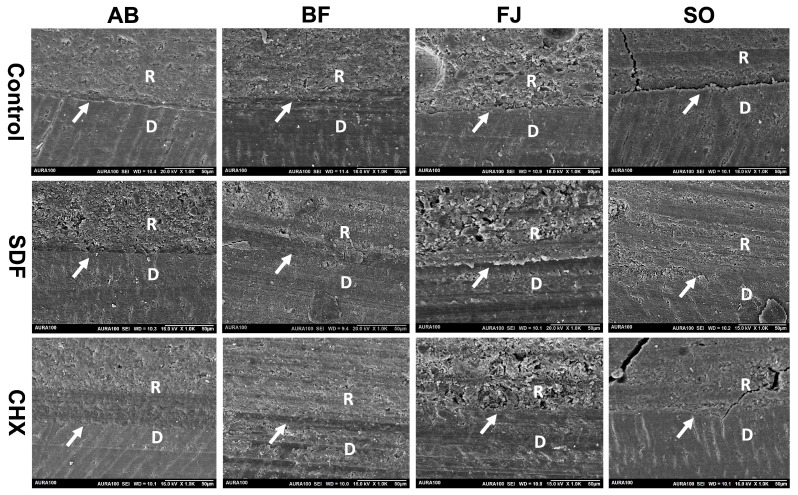
SEM images of the adhesive interface in different test groups (arrows indicate the interface, R represents restorative material, and D indicates the dentin surface).

Representative SEM images showing the interfacial bonding of different restorative materials with different surface treatments are shown in Figure 5. The control groups of AB and BF presented better interfacial morphology; the adhesive interfaces were uniform and well-defined, which did not significantly change after SDF or CHX treatment. However, the interface of FJ was slightly irregular. SO also displayed a limited and less homogenous interfacial adaptation to the underlying dentin. Nevertheless, SDF treatment resulted in an improved and more homogenous interface of both FJ and SO groups compared to their respective controls. 

## 4. Discussion

In this study, the bond strength of bioactive restorative materials was measured and evaluated after treating the dentin surface with two antibacterial agents: SDF and CHX. Four different commercially available restorative materials were tested in this study. Laboratory bond strength tests are often used to predict the clinical performance of newly introduced dental restorative materials and evaluate the effect of different operative variables. Shear testing was adopted in order to measure bond strength in this study for its convenient advantages and to overcome the limitation of a lower bond strength in some of the tested materials. 

In the control groups, AB and BF had a significantly higher bond strength compared to other tested materials. This may be attributed to the use of a bonding agent before material application, as illustrated in the manufacturer’s instructions for both materials. In fact, when AB was placed without adhesives in a pilot study, all restorations were separated immediately after specimen fabrication or after 24 h of storage, and measurement was not possible. 

The Single Bond Universal bonding agent was selected to bond AB and BF to the dentin. This adhesive contains both methacrylate-modified polyalkenoic acid copolymer (PAC) (also known as Vitrebond copolymer; 3M ESPE) and 10-methacryloyloxydecyl dihydrogen phosphate (10-MDP), which react chemically with hydroxyapatite in dentin [37]. The latter interaction has been shown to create a stable nanolayer with deposits of calcium-10 MDP salt at the dentin–adhesive interface, improving bond strength and durability [37,38]. Additionally, the enhanced resin monomer content in AB and BF may have facilitated copolymerization with the adhesive. The higher bond strength values recorded for the two groups were associated with a higher percentage of mixed and cohesive failures. 

BF is a light-cure giomer-based composite restorative system that uses modified surface-reacted prereacted glass ionomer particles (SPRGs) in a methacrylate-based resin. BF displayed SBS values that were higher than those of AB. This may be attributed to the resin monomers and the distinctive filler content. While monomers maintained mechanical strength and ensured effective copolymerization with the adhesive, the large-sized prepolymerized and SPRG fillers forming around 83.3 wt% also possibly increased SBS [28]. Additionally, the acid–base reaction of S-PRG fillers occurred during the fabrication process, which was reported to result in a surface-modified layer that protected the glass core [28]. The material also demonstrated a low level of volumetric shrinkage (0.85%) and a polymerization shrinkage stress of 2.72 MPa, as indicated by the manufacturer. 

AB is a dual-cure resin with a modified bioactive resin matrix and reactive glass ionomer fillers with mechanical properties that are claimed to be superior to those of RMGI and comparable to flowable and bulk-fill resin composites [28]. It is composed of 55.4 wt% bioactive glass fillers, methacrylate and diurethane monomers, polyacrylic acid, and modified diurethane dimethacrylate. Like RMGI, it has three hardening mechanisms: chemical reaction, light polymerization, and acid–base reaction [25]. It was reported to have a modulus of elasticity of 2.3 GPa, which was attributed to the patented rubberized resin component, and may have reduced SBS compared to BF [26].

In this study, RMGI FJ showed a weaker bond strength and did not significantly differ from SO. Most interfacial surfaces of these two groups represented adhesive interfacial failure. The RMGI adhesion mechanism is based on the shallow hybridization of the smear layer combined with the ionic interaction of carboxyl groups in polyacrylic acid with calcium in dentin hydroxyapatite [39,40]. SO has been recently introduced as a dual-cure bulk-fill hybrid restorative material with presumed self-adhesive and bioactive properties. In addition to the GIC adhesion mechanism, ionic interactions between calcium in dentin and the carboxyl groups of the modified polyacid (MOPOS) in SO have been demonstrated. MOPOS infiltrates the smear layer and partially demineralizes the underlying dentin, contributing to the adhesion to dentin [26].

In this study, the application of SDF reduced the bonding of BF and AB to dentin but enhanced that of FJ and SO and decreased adhesive failure in their debonded specimens. SBS values differed significantly in FJ. SDF applied to dentin reacted with hydroxyapatite, producing calcium fluoride (CaF_2_), silver phosphate (Ag_3_PO_4_), and ammonium hydroxide (NH_4_OH), which precipitated on the dentin surface and in dentin tubules, limiting monomer infiltration [41]. The chemical reaction mechanism between SDF and tooth structure was previously described [14]. Briefly, silver ions and the hydroxyapatite of the tooth structure react to form silver phosphate and calcium fluoride, followed by the dissociation of fluoride and calcium and the formation of fluorapatite. Additionally, previous studies reported a dense layer of silver phosphate on the dentin surface observed under SEM and the chemical bonding of SDF with the hydroxyapatite using Fourier transform infrared spectroscopy (FTIR) [42].

As the bonding mechanism of adhesives is based on micromechanical retention and hybrid layer formation, reduced bond strength is expected, especially since the adhesive was applied in self-etching rather than total-etching mode [37]. Etching with phosphoric acid was suggested to remove some precipitate SDF and therefore recover the bond strength [31]. The high pH of SDF could also hamper the etching function of the self-etching adhesive, reducing the bond strength [32]. Furthermore, SDF was applied according to the manufacturer’s instructions, and with rinsing after application. Previous studies reported a significant increase in bond strength via rinsing, as it eliminates the negative effect of SDF precipitates on the adhesive [33]. The improvement in the SBS of RMGI following the application of SDF is in accordance with the results of previous studies [40]. A previous study also reported that silver ion precipitates strengthen the ionic bond to GIC and enhance bond strength to dentin [43]. An increase in bond strength with SDF treatment was attributed to the chemical bond between the carboxylic acid of RMGIC with silver phosphate formed after the reaction between the tooth surface and SDF [44]. Moreover, SDF precipitates are thought to increase interfacial hardness and roughness at the GIC–dentin interface [43]. These results are in agreement with the SEM results of our study, as the FJ- and SO–SDF-treated groups showed an improved interfacial bond compared to their control groups. Moreover, these results were supported in our mode-of-failure results, as the FJ and SO groups predominantly displayed adhesive failure in the control group, which was improved following surface treatment with either CHX or SDF. 

In this study, CHX treatment did not significantly affect the bonding of the adhesive Single Bond Universal. A study by Nishitani et al. confirmed that 2% CHX does not reduce the degree of conversion of resin monomers [45]. Previous studies reported the positive effect of CHX in bond preservation both in the short and long term [16,46]. However, this effect on adhesive performance was reported to be type-dependent, as other studies reported a decrease in bond strength when self-etching adhesives were used [18,20].

The higher SBS of FJ treated with CHX could possibly be attributed to chemical changes caused by CHX application that increased the surface energy of dentin, which could result in higher wettability via RMGI [17,19]. On the other hand, CHX decreased the SBS of SO. This was speculated to be caused by the water content of CHX. Residual moisture after drying CHX might have interfered with the bonding mechanism and the maturation reaction of SO. In addition, CHX has strong cationic properties and may have reacted with the anionic carboxyl groups in MOPOS of SO, reducing its dentin-bonding capability [19].

One limitation of this in vitro investigation is that only immediate SBS was assessed, and no artificial aging was conducted to imitate the in vivo environment. When performing bond strength experiments, it is recommended to use degradation techniques, such as thermocycling, water storage, and fatigue stress, whenever possible, as some adhesive materials with high immediate SBS may show a decrease in bond strength after aging. Further research will be conducted to address this limitation.

## 5. Conclusions

Dentin bond strength varied across bioactive restorative materials and surface treatments. Bioactive materials placed in conjunction with bonding agents demonstrated better bonding performance than self-adhesive materials, regardless of the surface treatment. Treating dentin surfaces with antibacterial agents also did not interfere with the bond strength of self-adhesive bioactive materials. However, SDF treatment improved the bonding of RMGI. Therefore, the appropriate selection of surface treatment for each bioactive restorative material should be considered for efficient and minimally invasive treatment.

## Figures and Tables

**Table 1:** Materials used in this study and their composition and application methods.

Material, Manufacturer	Composition	Application Method
Advantage arrest, silver diamine fluoride 38%, Elevate Oral Care	Silver fluoride, ammonia, and deionized water	Dry the surface, apply, allow to soak in for 60 s, rinse, and dry.
Gluco-Chex 2%, Cerkamed, Stalowa Wola, Poland	Chlorhexidine digluconate 2% and water	Apply for 20 s, and air dry for 5 s.
Single Bond Universal, 3M, St. Paul, MN, USA	MDP, dimethacrylate, HEMA, Vitrebond copolymer, fillers, ethanol, water, initiator, and saline	Apply and rub for 20 s, gently air dry for 5 s, and light cure for 10 s.
Activa Bioactive Restorative, Pulpdent Corp., Watertown, MA, USA	Methacrylates, diurethane, silica, modified polyacrylic acid, and sodium fluoride	Bulk fill, allow to self-cure for 2 min, and light cure for 20 s.
Beautifil II, Shofu Corp., Tokyo, Japan	Bis-GMA, TEGDMA, aluminum oxide, aluminofluoro-borosilicate glass filler, prereacted glass ionomer filler, silica, and camphorquinone	Place a layer of 2 mm thickness, and light cure for 10 s.
Fuji II LC, GC Corp., Tokyo, Japan	Fluoroaluminosilicate, methacrylate, hydroxyethyl, polyacrylic acid, and water	Activate capsule, place in a mixer for 10 s, extrude paste, and light cure for 20 s.
Surefil One, Dentsply Sirona, Konstanz, Germany	Aluminum-phosphor-strontium- sodium-fluoro-silicate glass, water, silicon dioxide, acrylic acid, polycarboxylic acid, ytterbium fluoride, bifunctional acrylate, self-cure initiator, pigments, camphorquinone, and stabilizer	Activate capsule, place in a mixer for 10 s, extrude paste, and light cure for 20 s.

Abbreviations: MDP: 10-methacryloyloxydecyl dihydrogen phosphate; HEMA: 2-hydroxyethyl methacrylate; Bis-GMA: bisphenol A diglycidyl ether dimethacrylate; TEGDMA: trimethylene glycol dimethacrylate.

**Table 2:** Comparison of the shear bond strength (SBS) of individual materials between surface treatment groups.

Material	Control (n = 40)		SDF (n = 40)		CHX (n = 40)		*p*-Value ^	Multiple Comparisons #
	Mean (SD)	Median (IQR)	Mean (SD)	Median (IQR)	Mean (SD)	Median (IQR)		
AB	15.9 (4.4)	15.2 (12.3, 19.0)	13.8 (5.3)	13.2 (10.7, 15.8)	15.8 (6.4)	15.8 (11, 23.0)	0.555	Control vs. SDF Control vs. CHX SDF vs. CHX
BF	18.2 (6.0)	19.1 (15.0, 23.5)	12.7 (7.1)	11.7 (8.1, 14.6)	19.0 (11.3)	14.8 (11.5, 26.7)	0.212	Control vs. SDF Control vs. CHX SDF vs. CHX
FJ	4.0 (3.5)	2.7 (1.5, 5.3)	9.5 (3.7)	8.9 (6.6, 12.2)	7.0 (4.5)	5.4 (4.0, 8.4)	0.007	Control vs. SDF * Control vs. CHX SDF vs. CHX
SO	4.3 (3.3)	4.3 (2.2, 5.4)	8.2 (4.8)	8.4 (3.6, 10.7)	3.1 (1.6)	2.7 (2.2, 4.2)	0.031	Control vs. SDF Control vs. CHX SDF vs. CHX *
All materials	10.6 (7.8)	10.8 (3.3, 16.4)	11.0 (5.6)	10.7 (6.6, 13.9)	11.2 (9.3)	8.5 (4.1, 16.6)	0.724	Control vs. SDF Control vs. CHX SDF vs. CHX

* ^ Kruskal–Wallis test. # Wilcoxon rank-sum test. Significant differences (*p* ≤ 0.05) are marked with an asterisk.

**Table 3:** Comparison of SBS of the different materials within surface treatment groups.

Surface Treatment	AB (n = 30)		BF (n = 30)		FJ (n = 30)		SO (n = 30)		*p*-Value ^	Multiple Comparisons #
	Mean (SD)	Median (IQR)	Mean (SD)	Median (IQR)	Mean (SD)	Median (IQR)	Mean (SD)	Median (IQR)		
Control	15.9 (4.4)	15.2 (12.3, 19.0)	18.2 (6.0)	19.1 (15.0, 23.5)	4.0 (3.5)	2.7 (1.5, 5.3)	4.3 (3.3)	4.3 (2.2, 5.4)	<0.001	AB vs. BF AB vs. FJ * AB vs. SO * BF vs. FJ * BF vs. SO * FJ vs. SO
SDF	13.8 (5.3)	13.2 (10.7, 15.8)	12.7 (7.1)	11.7 (8.1, 14.6)	9.5 (3.7)	8.9 (6.6, 12.2)	8.2 (4.8)	8.4 (3.6, 10.7)	0.103	AB vs. BF AB vs. FJ AB vs. SO BF vs. FJ BF vs. SO FJ vs. SO
CHX	15.8 (6.4)	15.8 (11, 23.0)	19.0 (11.3)	14.8 (11.5, 26.7)	7.0 (4.5)	5.4 (4.0, 8.4)	3.1 (1.6)	2.7 (2.2, 4.2)	<0.001	AB vs. BF AB vs. FJ * AB vs. SO * BF vs. FJ * BF vs. SO * FJ vs. SO *
All treatments	15.2 (5.3)	14.3 (11.2, 19.0)	16.6 (8.6)	14.8 (10.6, 24.0)	6.8 (4.4)	5.6 (3.8, 9.5)	5.2 (4.0)	3.9 (2.4, 6.5)	<0.001	AB vs. BF AB vs. FJ * AB vs. SO * BF vs. FJ * BF vs. SO * FJ vs. SO

* ^ Kruskal–Wallis test. # Wilcoxon rank-sum test. Significant differences (*p* ≤ 0.05) are marked with an asterisk.

## Data Availability

Not applicable.
